# Dangers from Santonine

**Published:** 1877-01

**Authors:** 


Dangers from Santonine.—In using santonine it is well to bear in mind that comparatively small doses have produced convulsions of a somewhat grave character. A German contemporary lately reported a case in which poisonous effects were produced in a child two years old, by the ingestion of so small a dose as a grain and a half. Convulsions commenced in the face, and extended to the extremities, while the respiratory action was greatly impeded. Under warm baths, enemata, and artificial respiration, the patient recovered. The physician in charge of the case then instituted a series of experiments on the lower animals, and found that chloral and ether inhalations controlled the convulsions produced by santonine. He naturally argues that the same treatment should be pursued in the human subject when a poisonous dose is taken.—*Medical and Surgical Reporter.*

